# Effects of environment on human cytokine responses during childhood in the tropics: role of urban versus rural residence

**DOI:** 10.1186/s40413-015-0071-2

**Published:** 2015-08-06

**Authors:** Philip J. Cooper, Leila D. Amorim, Camila A. Figueiredo, Renata Esquivel, Fernanda Tupiza, Silvia Erazo, Yisela Oviedo, Maritza Vaca, Martha E. Chico, Mauricio L. Barreto

**Affiliations:** Centro de Investigación en Enfermedades Infecciosas y Cronicas, Facultad de Ciencias Exactas y Naturales, Pontifícia Universidad Católica Del Ecuador, Quito, Ecuador; Colegio de Ciencias de la Salud, Universidad San Francisco de Quito, Quito, Ecuador; Laboratorio de Investigaciones FEPIS, Quininde, Esmeraldas Province Ecuador; Institute of Infection and Immunity, St. George’s University of London, Cranmer Terrace, Tooting, London, SW17 ORE UK; Instituto de Matemática, Universidade Federal de Bahia, Salvador, Brazil; Instituto de Ciências da Saúde, Universidade Federal de Bahia, Salvador, Brazil; Instituto de Saúde Coletiva, Universidade Federal de Bahia, Salvador, Brazil

**Keywords:** Environment, Hygiene, Cytokine profile, Rural, Urban, Tropical Latin America

## Abstract

**Background:**

Environment may have a key role in the development of the immune system in childhood and environmental exposures associated with rural residence may explain the low prevalence of allergic and autoimmune diseases in the rural tropics. We investigated the effects of urban versus rural residence on the adaptive immune response in children living in urban and rural areas in a tropical region of Latin America.

**Methods:**

We recruited school children in either rural communities in the Province of Esmeraldas or in urban neighborhoods in the city of Esmeraldas, Ecuador. We collected data on environmental exposures by questionnaire and on intestinal parasites by examination of stool samples. Peripheral blood leukocytes (PBLs) in whole blood were stimulated with superantigen, parasite antigens and aeroallergens and IFN-γ, IL-5, IL-10, IL-13, and IL-17 were measured in supernatants.

**Results:**

We evaluated 440 school children; 210 living in rural communities and 230 in the city of Esmeraldas. Overall, urban children had greater access to piped water (urban 98.7 % vs. rural 1.9 %), were more likely to have a household bathroom (urban 97.4 % vs. rural 54.8 %), and were less likely to be infected with soil-transmitted helminth infections (urban 20.9 % vs. rural 73.5 %). Generally, detectable levels of cytokines were more frequent in blood from children living in urban than rural areas. Urban residence was associated with a significantly greater frequency of IL-10 production spontaneously (adjusted OR 2.56, 95 % CI 1.05-6.24) and on stimulation with *Ascaris* (adj. OR 2.5, 95 % CI 1.09-5.79) and house dust mite (adj. 2.24, 95 % CI 1.07-4.70) antigens. Analysis of effects of environmental exposures on SEB-induced IL-10 production within urban and rural populations showed that some environmental exposures indicative of poor hygiene (urban – higher birth order, *A. lumbricoides* infection; rural - no bathroom, more peri-domiciliary animals, and living in a wood/bamboo house) were associated with elevated IL-10.

**Conclusions:**

In our study population, the immune response of children living in an urban environment was associated more frequently with the production of the immune regulatory cytokine, IL-10. Some factors related to poor hygiene and living conditions were associated with elevated IL-10 production within urban and rural populations.

**Electronic supplementary material:**

The online version of this article (doi:10.1186/s40413-015-0071-2) contains supplementary material, which is available to authorized users.

## Background

Rural residence has been shown in several epidemiologic studies to be associated with a reduced prevalence of allergic sensitization and allergic diseases including asthma [1]. The protective effect of rural living has been associated with farm [2, 3] and other exposures in early life indicative of an increased exposure to microbes and their products [4, 5]. In the rural tropics, there has been considerable interest in the protective role against allergy of chronic parasitic infections such as soil-transmitted helminths (STH), that are highly prevalent during childhood, and that have been shown to be associated with reduced atopy [6, 7] but with variable effects on asthma prevalence [8].

Intense exposures during early life to microbes, parasites, and their products may play an important role in educating the immune system to respond to different stimuli with an appropriate but measured immune response. Particularly important may be the development of a robustly regulated immune response in which potentially harmful pathogens are dealt with appropriately but responses to innocuous antigens such as aerollergens or those of intestinal microbiota are tightly regulated to prevent potentially damaging inflammation.

There are few published data comparing the immune responses of children between urban and rural populations in the tropics. An understanding of immune responses in different environments, and how specific environmental exposures may affect this response, is relevant for our understanding of why inflammatory diseases including allergy and autoimmunity may be increasing in urban and urbanizing populations in low and middle income countries [9, 10]. Previous studies done in urban Brazil have provided evidence that environmental exposures associated with poor hygiene favor the development of stronger immune regulation [11, 12]. Further, children living in rural Africa had altered innate immunity and impaired vaccine immune responses [13] compared to those living in an urban environment, effects that were attributed to the immunologic effects of chronic helminth infections [14].

To test the hypothesis that rural residence and environmental exposures suggestive of a greater exposure to infections and microbes would be associated with alterations in cytokine responses and increased immune regulation, we compared cytokine responses between school children living in remote rural communities with those of children living in the capital of the same Province. Cytokine responses were measured in peripheral blood leukocytes (PBLs) in whole blood cultured in the presence of a variety of adaptive immune stimuli. To understand better how environment may affect the human cytokine response, we examined the effects of area of residence and individual environmental exposures on cytokine responses.

## Materials and methods

### Study design and population

We analysed data collected from two nested case–control studies of wheezing illness done in school-age children in rural communities in the Districts of Eloy Alfaro and San Lorenzo, Esmeraldas Province and in neighborhoods in the city of Esmeraldas that had a high proportion of migrants from the same two rural Districts. The design of this study has been described in detail elsewhere [15]. Briefly, subjects were selected using data from previous cross-sectional studies done in the same rural and urban areas. Cases were defined as children with wheeze within the previous 12 months and controls as children without wheeze ever. Detailed information on wheeze symptoms, demographic and lifestyle factors was obtained by parental questionnaire.

### Collection of blood and stool samples

Single stool samples were collected and examined for the presence of helminth eggs and larvae using the modified Kato–Katz and the formol-ethyl acetate-concentration methods. Blood samples (3 mL) were collected into plastic tubes (BD Vaccutainer, Plymouth, UK) containing sodium heparin as an anticoagulant.

### Whole blood culture

Heparinized blood was diluted 1:4 in RPMI 1640 medium (BioWhittaker, Walkersville, MD) containing l-glutamine (BioWhittaker), 80 mg/mL gentamicin and 1 % HEPES (GIBCO BRL, Gaithersburg, MD). Diluted whole blood (0.5 mL) was cultured alone or in the presence of *A. lumbricoides* adult worm antigen prepared as described previously [16] at 10 μg/mL, *D. pteronyssinus* (glycerinated solution, standardized at 5,000 AU/mL; Greer Laboratories, Lenoir, NC, USA) at a dilution of 1/50, and the superantigen, *Staphylococcus* enterotoxin B (SEB; Sigma Aldrich, St Louis, MO, USA) at 1 μg/mL. The cells were cultured within 5 h of collection and were maintained in a humidified environment of 5 % CO_2_ at 37 °C for 5 days for the detection of cyokines.

### Cytokine assays

We measured the production of Th2 (IL-5 and IL-13), Th1 (IFN-γ), Th17 (IL-17) and Treg (IL-10) cytokines in whole blood culture supernatants using commercially available antibody pairs and recombinant cytokine standards (BD Pharmingen Biosciences, CA, USA) by sandwich ELISA according to the manufacturer's instructions. Cytokine concentrations were determined by interpolation of standard curves. Responders were defined as those children with cytokine concentrations above the lower detection limits: for IFN-γ (8 pg/mL), IL-13 (144 pg/mL), IL-5 (20 pg/mL), IL-10 (4 pg/mL), and IL-17 (2 pg/mL). Values below this limit were regarded as non-responders.

### Statistical analyses

Comparisons of socio-demographic and environmental variables by residence (urban vs. rural) were done using chi-square test or Fisher exact test, as appropriate. Cytokine production for each stimulus (spontaneous [media alone], SEB, *Ascaris lumbricoides*, house dust mite) was expressed using the median and inter-quartile range. Cytokine responder rates were the proportions of children with cytokine concentrations above the assay threshold. To measure the associations between area of residence and cytokine responders, logistic regression models were fitted considering three sets of covariates that could influence this relationship: Model 1 included the child’s age and sex as covariates; Model 2 included age, sex, maternal education, type of bathroom in household and presence of infection with *Giardia lamblia*; Model 3 included age, sex, maternal education, type of bathroom in household and soil-transmitted helminth (STH) infections (*Ascaris lumbricoides* or *Trichiura trichiura*). These three sets of covariates were chosen based on results of univariate analyses and previous published findings [11, 12, 17]. Where variables were highly correlated, those with greater predictive power for outcomes were included. Effects of area of residence and environmental exposures on SEB-induced cytokine responses were explored using linear regression in which we built models that included the three sets of covariates. Results of linear regression were back-transformed to provide fold-change in geometric means. P < 0.05 was considered statistically significant. Statistical analyses were done using STATA (version 10) software.

### Ethical considerations

Informed written parental consent was obtained for all children. The ethics committees of the Universidad San Francisco de Quito, Quito, and Hospital Pedro Vicente Maldonado, Pichincha Province, Ecuador, approved the study protocol.

## Results

### Difference in environmental and other factors between urban and rural populations

There were a number of differences with respect to socio-demographic factors between children in the urban compared to the rural study samples (Table 1): urban children were younger (P < 0.001), were less likely to be Afro-Ecuadorian (P < 0.001), and had better-educated mothers (P < 0.001). Urban children had a lower median BMI than rural children (P < 0.001) but this may be explained by their lower median age. We classified environmental exposures into those directly associated with hygiene, farming, and with specific enteric parasite infections. With respect to hygiene exposures, children living in rural households were more likely to live in conditions associated with poorer hygiene and with a higher risk of infections: being lower in the birth order (i.e. have more older siblings) (P = 0.02), having attended daycare (P < 0.001), not having access to potable drinking water (P < 0.001), and living in traditionally-constructed houses made from wood and bamboo (P < 0.001) compared to urban children. Similarly, rural children were more likely to have farming exposures than urban children: having a father with an agricultural occupation (P < 0.001), being exposed to a wider variety of animals around the house (P < 0.001), and having contact with large farm animals (P < 0.001). With respect to enteric parasite infections, rural children were more likely to be infected with STH parasites (P < 0.001) and *Entamoeba histolytica/dispar* (P = 0.005), but less likely to have *Giardia intestinalis* cysts (P = 0.04) in stool samples compared to urban children. Urban children were more likely to have received anthelmintic treatment within the previous 12 months (P < 0.001).

**Table 1:** Distributions of environmental and socio-economic characteristics by area of residence for study subjects

Characteristic	Rural	Urban	P value*
	(N = 210)	(N = 230)	
**Socio-demographic**			
**Age (years)**			
Median (range)	11 (7–18)	9 (6–14)	<0.001
**Sex**			
Male/female (%)	56.2/43.8	48.5/51.5	0.106
**Ethnicity**			
Afro-Ecuadorian/Mestizo (%)	97.1/2.9	74.2/25.8	<0.001
**Maternal educational level (%)**			
Illiterate	49.5	15.7	<0.001
Primary complete	43.8	47.7	
Secondary complete	6.7	36.6	
**Monthly income (US$)**			
Median (range)	195 (10–5,000)	220 (0–3,000)	0.072
**Body mass index**			
Median (range)	17.6 (16.1-20.0)	16.3 (15.5-18.1)	<0.001
**Environmental/hygiene**			
**Pets inside the house (%)§**	65.1	58.1	0.173
**Crowding (persons/sleeping room)**			
Median (range)	2.67 (2–4)	2.67 (2–4)	0.974
**Birth order**			
1^st^-2^nd^	37.1	51.6	0.020
3^rd^-4^th^	31.4	25.8	
≥5th	31.4	22.6	
**Bathroom (%)**			
Field	45.2	2.6	<0.001
Latrine	48.6	25.8	
WC	6.2	71.6	
**Daycare attendance (%)**	58.2	33.99	<0.001
Drinking water sources*			
Rain (%)	70.5	1.3	<0.001
River (%)	57.6	1.3	<0.001
Well (%)	31.9	1.3	<0.001
Piped (%)	46.7	0	<0.001
Potable (%)	1.9	98.7	<0.001
**House construction materials (%)**			
Wood/bamboo	58.3	26.5	<0.001
Wood/cement	18.7	27.7	
Cement/brick	23.0	45.8	
**Environmental/farming**			
**Father with agricultural occupation (%)**	75.2	47.7	<0.001
**Number of per-domiciliary animals†**			
0	5.7	5.8	<0.001
1-3	49.3	66.2	
≥4	45.0	26.0	
**Contact with large farm animals‡ (%)**	20.6	3.2	<0.001
**Environmental/infections (%)**			
Any geohelminth	73.5	20.9	<0.001
*A. lumbricoides*	50.0	9.6	<0.001
*T. trichiura*	62.0	16.8	<0.001
*G. intestinalis*	8.5	15.0	0.040
*E. histolytica/dispar*	27.0	15.9	0.005
Recent anthelmintic treatment (%)¶	66.0	85.6	<0.001

*P values calculated using the Chi-squared, Fisher’s exact test, or Mann–Whitney test as appropriate. **Households, especially in the rural area, have multiple sources of drinking water that vary by season. † − numbers of the following different animals: dog, cat, pig, chicken, cow, donkey, horse. § dog and or cat passing freely inside house. ‡ at least once weekly. ¶ - within previous 12 months

### Differences in cytokine responses between urban and rural children

Because there were no significant differences in PBL cytokine responses between wheezer case and non-wheezer control children in either the urban or rural area (Additional file 1: Table S1), cytokine data for cases and controls in each area were pooled into urban and rural responses. Table 2 shows the medians and inter-quartile ranges for each of the cytokines to the different culture stimuli and (Additional file 1: Figure S1a-e) shows these data graphically. All stimuli, with the exception of SEB, resulted in a highly skewed distribution of cytokine responses with most individuals not having detectable levels of cytokines greater than spontaneous (i.e. whole blood cultured in the presence of medium only). Therefore, cytokine data for SEB are shown as fold-changes in cytokine levels while the data for the other culture conditions as response rates. Response rates for cytokines produced by PBLs in the presence of medium (spontaneous), *A. lumbricoides*, and house dust mite antigens are shown in Additional file 1: Table S2. In logistic regression models that controlled for age and sex, we observed a higher prevalence of responders producing IL-13 (adjusted OR 1.54, 95 % CI 1.01-2.37), IFN-γ (adj. OR 1.88, 95 % CI 1.19-2.98), and IL-10 (adj. OR 1.86, 95 % CI 1.05-3.32) spontaneously among urban compared to rural children (Table 3). A similar trend was seen for IL-5 (adj. OR 1.42, 95 % CI 0.93-2.15) but not for IL-17. We also observed an increased frequency of urban children responding to antigen with the production of IL-10 (*A. lumbricoides* antigen, adj. OR 1.83, 95 % CI 1.08-3.11; house dust mite, adj. OR 2.42, 95 % CI 1.54-3.78) and IL-5 (house dust mite, adj. OR 1.73, 95 % CI 1.06-2.82) compared to rural children. STH infections have been identified in previous studies as key mediators of increased production of IL-10 and Th2 cytokines by PBLs [18]. To explore the potential contribution of STH infections to the increased production of IL-10 and Th2 cytokines observed in this study, we fitted multivariate models to determine whether STH infections in contrast to another non-helminth enteric parasite infection, *Giardia intestinalis*, might mediate the effects of residence on cytokine production. To do this, we constructed logistic regression models in which we controlled for the effects of the potential intermediate variables (STH infections or *G. intestinalis*). An intermediate variable would be expected to reduce the OR towards 1.00. These models were also controlled for maternal education and type of bathroom that were strongly associated with these enteric parasitic infections (data not shown). For IL-10, controlling for the effects of either *G. intestinalis* (Model 2) or STH infections (Model 3) did not reduce, but actually increased the ORs, indicating that these variables did not mediate but rather confounded the effects. In the case of cytokine production in response to SEB stimulation, we examined the effect of residence on the fold-change in cytokine levels (Table 4). There was some evidence for an increased production of IL-10 among urban compared to rural children after controlling for either *G. intestinalis* or STH infections, although these effects were not statistically significant. Controlling for both *G. intestinalis* and STH infections did not materially affect ORs for responder rates or fold-changes in cytokine levels (data not shown).

**Table 2:** Production of Th2 (IL-5 and IL-13), Th1 (IFN-γ), T reg (IL-10) and Th17 (IL-17) cytokines by peripheral blood leukocytes exposed to different culture conditions stratified by rural vs. urban residence

Cytokine Production	Spontaneous	SEB*	Ascaris	HDM
	M_d_(Q_1_-Q_3_)	M_d_(Q_1_-Q_3_)	M_d_(Q_1_-Q_3_)	M_d_(Q_1_-Q_3_)
**Rural Area**				
**IL-5**	70 (41–154)	2940 (2126–4452)	76 (21–228.5)	17 (9–37)
**IL-13**	269 (202–475)	4511 (3585–6125)	49 (18–128)	30 (14–61)
**IFN-γ**	86 (34–250)	638 (285–2450)	61 (18–132)	49 (15–131)
**IL-10**	14 (8–39)	377 (239–588)	12 (7–28)	25 (7–62)
**IL-17**	16 (9–40)	327 (256–419)	13 (7–17)	15 (6–21)
**Urban Area**				
**IL-5**	67 (41–152)	2692 (1900–3992)	53 (19–141)	19 (8–56)
**IL-13**	321 (213–564)	4148 (3146–5531)	44 (16–121)	35 (14–62)
**IFN-γ**	169 (68–373)	391 (214–873)	63 (22–128)	46 (10–177)
**IL-10**	16 (8–52)	392 (268–591)	15 (6–35)	25 (10–60)
**IL-17**	11 (7–55)	335 (231–490)	8 (4–23)	14 (4–23)

*SEB- *Staphylococcus* enterotoxin B

**Table 3:** Associations between area of residence (urban vs. rural) and cytokine production for each culture condition in which different models represent adjustments for different sets of variables

Stimulus/Cytokine	Model 1	Model 2	Model 3
	OR (95%CI)	OR (95%CI)	OR (95%CI)
**Spontaneous production**			
**IL-5** (n = 418)	1.42 (0.93;2.15)	1.00 (0.52;1.92)	0.95 (0.48;1.87)
**IL-13** (n = 418)	**1.54 (1.01;2.37)**	0.67 (0.34;1.32)	0.53 (0.26;1.07)
**IFN-γ** (n = 417)	**1.88 (1.19;2.98)**	1.67 (0.83;3.37)	1.50 (0.72;3.10)
**IL-10** (n = 415)	**1.86 (1.05;3.32)**	**2.64 (1.12;6.22)**	**2.56 (1.05;6.24)**
**IL-17** (n = 416)	0.83 (0.37;1.84)	2.13 (0.66;6.84)	1.61 (0.48;5.42)
***Ascaris lumbricoides***			
**IL-5** (n = 418)	0.75 (0.48;1.15)	1.50 (0.75;3.00)	1.53 (0.74;3.15)
**IL-13** (n = 418)	0.66 (0.44;1.01)	1.14 (0.59;2.22)	1.29 (0.65;2.57)
**IFN-γ** (n = 417)	1.35 (0.78;2.33)	1.73 (0.72;4.12)	1.67 (0.67;4.14)
**IL-10** (n = 416)	**1.83 (1.08;3.11)**	**2.43 (1.09;5.38)**	**2.51 (1.09;5.79)**
**IL-17** (n = 416)	0.69 (0.33;1.47)	0.79 (0.27;2.34)	0.61 (0.19;1.95)
**House dust mite**			
**IL-5** (n = 418)	**1.73 (1.06;2.82)**	2.06 (0.98;4.29)	2.09 (0.97;4.52)
**IL-13** (n = 418)	0.89 (0.58;1.35)	1.54 (0.80;2.99)	1.48 (0.74;2.94)
**IFN-γ** (n = 417)	1.45 (0.85;2.46)	1.63 (0.68;3.92)	1.57 (0.64;3.87)
**IL-10** (n = 416)	**2.42 (1.54;3.78)**	1.83 (0.91;3.68)	**2.24 (1.07;4.70)**
**IL-17** (n = 411)	1.22 (0.57;2.61)	1.63 (0.61;4.38)	1.63 (0.57;4.67)

Model 1 adjusted for age and sex

Model 2 adjusted for age, sex, Giardia, maternal education and bathroom

Model 3 adjusted for age, sex, worm infection (*Ascaris* and or *Trichuris*), maternal education and bathroom

Odd ratios (OR) calculated using logistic regression

ORs in bold with P < 0.05

**Table 4:** Associations between area of residence and cytokine production by superantigen (SEB)- stimulated peripheral blood leukocytes

Cytokines	Model 1	Model 2	Model 3
	Fold-change	Fold-change	Fold-change
	(95%CI)	(95%CI)	(95%CI)
**IL-10**	0.83 (0.69;1.01)	1.27 (0.96;1.69)	1.30 (0.97;1.75)
**IL-17**	0.96 (0.83;1.11)	1.19 (0.95;1.47)	1.19 (0.95;1.49)
**IFN-γ**	0.84 (0.60;1.18)	0.82 (0.48;1.41)	0.82 (0.47;1.42)
**IL-13**	0.85 (0.74;0.97)	1.14 (0.92;1.41)	1.20 (0.96;1.50)
**IL-5**	0.80 (0.67;0.95)	0.92 (0.71;1.19)	1.00 (0.76;1.31)

Fold-changes represent ratios of geometric means using linear regression and are adjusted for different sets of variables as follows: Model 1 - age and sex

Model 2 - age, sex, Giardia, maternal education and bathroom; Model 3 - age, sex, worm infection (*Ascaris* and or *Trichuris*), maternal education and bathroom

95 % CI – 95 % confidence intervals

SEB- *Staphylococcus* enterotoxin B

### Effects of individual environmental exposures on IL-10 production

We explored which environmental exposures might mediate the effects of urban residence on IL-10 production, a cytokine of particular interest because of its immune modulatory effects [19], and which was significantly increased for all stimuli (Table 3, Model 3). To do this, we evaluated the associations between individual environmental exposures and SEB-induced production of IL-10 within each of the urban and rural study samples (Table 5). We observed effects of environmental exposures on increased IL-10 production that were distinct for urban and rural samples. In the urban sample, these exposures were being higher in the birth order, being infected with *A. lumbricoides*, but not having a father with an agricultural occupation, while in the rural sample, these exposures were living in a wood/bamboo house (compared to a cement-brick house) and having a greater number of peri-domiciliary animals. None of the individual environmental exposures were significantly associated with response rates for IL-10 for the other culture conditions (data not shown).

**Table 5:** Associations between environmental factors and IL-10 production by superantigen (SEB)-stimulated peripheral blood leukocytes stratified by area of residence

Environmental factor	Urban	Rural
	Fold-change (95 % CI)	Fold-change (95 % CI)
**Environmental/hygiene**		
Pets inside the house		
No	1	1
Yes	1.24 (0.77;1.35)	1.00 (0.76;1.31)
Crowding (persons/sleeping room)		
≤ median	1	1
> median	0.90 (0.69;1.18)	1.07 (0.82;1.39)
Birth order		
1^st^-2^nd^	1	1
3^rd^-4^th^	**1.65 (1.21;2.26)**	0.99 (0.73;1.36)
≥5^th^	**1.66 (1.20;2.32)**	1.10 (0.80;1.52)
Bathroom		
Latrine	1	1
WC	0.80 (0.59;1.09)	0.79 (0.46;1.38)
Field	0.86 (0,36;2.06)	**1.34 (1,03;1.76)**
Daycare attendance		
No	1	1
Yes	0.79 (0.59;1.06)	1.07 (0.82;1.40)
Household construction		
Wood/bamboo	1	1
Wood/cement	1.21 (0.84;1.76)	1.03 (0.72;1.48)
Cement/brick	0.94 (0.67;1.30)	**0.68 (0.49;0.93)**
**Environmental/farming**		
Father with agricultural activities		
No	1	1
Yes	**0.76 (0,58;0.99)**	0.85 (0.63;1.14)
Number of peri-domiciliary animals†		
0	1	1
1-3	1.42 (0.76;2.68)	1.70 (0.97;3.01)
≥4	1.11 (0.59;2.07)	**1.91 (1.08;3.35)**
Contact with large farm animals‡		
No	1	1
Yes	0.57 (0.27;1.20)	1.06 (0.77;1.46)
**Environment/infections**		
Any geohelminth		
No	1	1
Yes	1.29 (0.58;1.75)	1.15 (0.85;1.56)
*A. lumbricoides*		
No	1	1
Yes	**1.67 (1.09;2.57)**	1.17 (0.90;1.53)
*T. trichiura*		
No	1	1
Yes	1.16 (0.83;1.61)	1.08 (0.82;1.43)
*G. intestinalis*		
No	1	1
Yes	0.76 (0.53;1.07)	1.16 (0.71;1.90)
*E. histolytica/dispar*		
No	1	1
Yes	**0.62 (0.44; 0.88)**	1.00 (0.74; 1.35)

Fold-changes represent ratios of geometric means using linear regression and are adjusted for age and gender

95 % CI – 95 % confidence intervals

SEB- *Staphylococcus* enterotoxin B

ORs in bold with P < 0.05

## Discussion

In this study we have examined the effects of area of residence and environment on cytokine responses by comparing Th1 (IFN-γ), Th2 (IL-5 and IL-13), Th17 (IL-17), and T regulatory (IL-10) cytokine responses to a range of immune stimuli in school age children living in urban and rural areas of Esmeraldas Province in a tropical coastal region of Ecuador. Specifically, we hypothesized that rural residence and environmental exposures indicative of greater exposures to infections and microbes in and around the household would be associated with greater immune regulation characterized by IL-10 responses. However, somewhat surprisingly, we observed that children living in urban areas had a significantly increased frequency of producing detectable levels of the immune regulatory cytokine, IL-10 spontaneously and in cultures stimulated with house dust mite (HDM) and *A. lumbricoides* antigen. Further there was a trend, albeit non-significant, of increased SEB-induced IL-10 production (used as a measure of maximal IL-10 production) in urban compared to rural children. When we tried to identify individual environmental exposures that might explain this finding, none of the exposures measured were significantly associated with IL-10 responder rates but some exposures that might be considered to reflect poorer hygiene (i.e. higher in birth order, greater exposure to domestic animals, and infections with *A. lumbricoides*) were associated with increased IL-10 production in the urban population. Our data, therefore, provide evidence that area of residence and the associated environment may have effects on the development of the human immune response and that some exposures associated with poor hygiene may partly mediate an increased production of the immune regulatory cytokine, IL-10.

Environment is considered to have a critical role in the development of inflammatory diseases, particularly allergy and autoimmunity [11]. The temporal trends of increased prevalence of allergic diseases such as asthma in industrialized countries over recent decades have been attributed to changes in the living environment. It has been suggested that a decline in exposures to infectious diseases during childhood, in particular, may have contributed to a failure in the development of robust immune regulation that may be required to regulate Th1, Th2, and Th17 inflammatory responses. A failure to regulate these responses may contribute to the development of allergic and autoimmune diseases. The development of immune regulation may not just be determined by pathogens but also by exposure to diverse microorganisms and their products present in the host (i.e. microbiota) and environment (e.g. animals, soil, and plants) and which have become altered in the modern environment.

There is evidence that the prevalence of inflammatory diseases may be greater in urban compared to rural populations [17, 20] and a explanation for this, at least in the context of the hygiene hypothesis, could be that environmental microbial exposures are greater in the rural environment leading to the development of adequate immune regulation during childhood thereby reducing the risk of inflammatory diseases. Such effects might be associated with reduced immune regulation (i.e. IL-10 production to a variety of immune stimuli) in urban compared to rural populations and there are some data that support this supposition [17, 21]. However, urban environments in the developing world are not necessarily more hygienic than rural environments, particularly among populations living in marginal urban neighborhoods (i.e. the *pueblos jovenes* or *favelas* of Latin America) under conditions of intense crowding and a lack of infrastructure (e.g. paved roads, clean water and sanitation) and services (e.g. rubbish disposal). A previous study done among children living in poor neighborhoods in urban Brazil has shown that factors associated with poor hygiene (i.e. a lack of paved roads, rubbish disposal and a municipal sewage connection) were associated with an increased frequency of IL-10 production by PBLs [11]. In the present study, we observed that children living in an urban compared to a rural environment were more likely to produce IL-10. This finding was unexpected – we had hypothesized that rural residence would be associated with greater IL-10 responder frequencies and cytokine production because of the poorer and presumably less hygienic living conditions of rural compared to urban schoolchildren. However, in a previous study comparing cytokine responses between infants aged 6–9 months in the same rural and urban environments, we observed also elevated production of SEB-induced IL-10 among the urban population [22]. These observations raise the question that if the mechanism underlying this hypothesis is correct - that less hygienic conditions contribute to immune regulation – then there are actually a limited number of exposures that may be critical for this effect and these exposures, unmeasured in the present study, were more common in the urban environment. The urban neighborhoods in the present study were selected by having a high proportion of migrants from the rural study Districts, were situated on the growing periphery of the City of Esmeraldas, and were characterized by a high level of poverty and limited access to services. One candidate exposure that might be relevant is the treatment of so-called potable water, used by almost all urban households, and which could be vulnerable to a high level of microbial contamination.

Although we could not identify environmental factors that were associated with IL-10 responder rates, there was some evidence that several factors associated with poor hygiene, and suggestive of greater exposures to infections, microbes, and microbial diversity, were associated with greater IL-10 production. These factors included being higher in the birth order (e.g. greater exposure to infections brought home by older siblings during early childhood), living in wood/bamboo house (e.g. exposure to natural microbiota from plants used for building or the environment associated with such constructions), being exposed to domestic animals (e.g. sources of faecal and related animal microbiota), and being infected with *A. lumbricoides* parasites in childhood. The environmental exposures associated with increased IL-10 production differed between rural and urban areas, a fact that might be explained by differences in power (i.e. infrequent exposures in either area were unlikely to have sufficient power for anything other than large effects to be demonstrated) or a lack of heterogeneity in exposure within areas. In the case of the latter, although exposure to *A. lumbricoides* in the rural area may be universal (e.g. as might be determined by the presence of parasite-specific IgG antibodies [16], point prevalence was just 50 %. Previous infections or light undetected infections among children classified as uninfected and the immunologic effects of those previous or undetected infections, could reduce substantially the discriminatory power of an analysis comparing infected versus uninfected children where infection status was determined at a single point in time. However, in the urban area with a much lower prevalence of infection, one could be more confident that a much higher proportion of uninfected children were truly uninfected both at the time of sampling and in the past, thus allowing an effect of *A. lumbricoides* infection to be more clearly demonstrated.

We observed that PBL cultures from urban schoolchildren were more likely to produce IL-10, IL-13, and IFN-γ constitutively (or spontaneously) than those from rural schoolchildren (model 1). However, the effects on IL-13 and IFN-γ disappeared after controlling for environmental factors, indicating that such factors might partly mediate the urban effect for these cytokines. In contrast, controlling for these factors did not affect the urban effect on IL-10 that was observed not just spontaneously but for all adaptive immune stimuli, indicating that other unmeasured factors may mediate the urban effect on IL-10. We have shown previously in an urban population in Brazil an association between poor hygiene exposures and increased IL-10 produced by PBLs spontaneously [11] and after mitogen stimulation [12], and that STH infections are important up-regulators of IL-10 in the urban environment [21]. Our observations of an increased frequency of IL-10 production among urban schoolchildren, and the observation within the urban sample that several poor hygiene exposures including *A. lumbricoides* infection were associated with increased IL-10 production, are broadly consistent with these observations.

There are several limitations that should be considered in the interpretation of our study findings. Firstly, although the urban and rural populations were selected to be broadly comparable with respect to ethnicity and source population, there were a large number of differences between urban and rural samples with respect to measured and presumably unmeasured confounders. Thus, we cannot exclude uncontrolled confounding as an alternative explanation for our findings. Similarly, much of the environmental data were collected by questionnaire and differences in the educational level of parents could lead to biases in data collection between urban and rural areas. Although, this was a relatively large study for the measurement of immunological outcomes, sample size was limited by logistic and cost considerations, and we therefore had limited power to detect small effects or for those of infrequent exposures. Finally, the analysed data were derived from case–control studies for wheezing illness in which data for cases and controls were pooled for each area of residence. Cytokine data did not differ between cases and controls, probably explained by the fact that wheezing illness in this population is mild, does not require maintenance treatment, and is only weakly associated with atopy [23]. This form of mild non-atopic illness likely explains the lack of differences in cytokine production between cases and controls, particularly with respect to Th2 cytokines.

## Conclusions

To our knowledge, this is the first study to explore differences in cytokine production by PBLs in response to a range of immunologic stimuli between urban and rural populations in the Tropics. We observed that urban schoolchildren were more likely to produce the immune regulatory cytokine, IL-10 in response to all immunologic stimuli, compared to their rural counterparts. Within the urban sample, several environmental exposures indicative of poor hygiene were associated with elevated SEB-induced production of IL-10. Our data, thus, provide only limited support for the hypothesis that poor hygiene and increased exposure to a microbially diverse environment contribute to immune regulation, and do not explain why IL-10 was produced less frequently by rural schoolchildren who presumably lived in a more microbially diverse environment. It is possible that one or a few critical exposures not measured in the present study and that mediate the immune regulatory effect, were more frequent or intense in poor neighborhoods of the crowded periphery of cities like the City of Esmeraldas where the present study was conducted.

## Additional file

Additional file 1: Table S1. Production of Th2 (IL-5 and IL-13), Th1 (IFN-g), Treg (IL-10), and Th17 (IL-17) cytokines according to wheezing status (cases and controls). **Table S2.** Responder rates by area of residence (urban vs. rural) and cytokine for each culture condition. **Figure S1a-e.** Production of cytokines by children living in urban and rural environments. a) IL-5, b) IL-13, c) IFN-g, d) IL-10, and e) IL-17.

## Abbreviations

STH: Soil-transmitted helminth
HEPES: 4-(2-hydroxyethyl)-1-piperazineethanesulfonic acid
