# Supplementary material for: Effects of environment on human cytokine responses during childhood in the tropics: role of urban versus rural residence
Source: World Allergy Organ J. 2015 Aug 6;8(1):22. doi: 10.1186/s40413-015-0071-2 (PMC4527255; doi:10.1186/s40413-015-0071-2)
Supplement: Additional file 1: Table S1. — Production of Th2 (IL-5 and IL-13), Th1 (IFN-g), Treg (IL-10), and Th17 (IL-17) cytokines according to wheezing status (cases and controls). Table S2. Responder rates by area of residence (urban vs. rural) and cytokine for each culture condition. Figure S1a-e. Production of cytokines by children living in urban and rural environments. a) IL-5, b) IL-13, c) IFN-g, d) IL-10, and e) IL-17. [file 40413_2015_71_MOESM1_ESM.docx]

**Table S1.** Production of Th2 (IL-5 and IL-13), Th1 (IFN-g), T reg (IL-10) and Th17 (IL-17) cytokines according to wheezing status (case and control)

| **Cytokine Production** | **Spontaneous** | **Ascaris** | **HDM** | **SEB** |
| --- | --- | --- | --- | --- |
|  | **M_d_(Q_1_-Q_3_)** | **M_d_(Q_1_-Q_3_)** | **M_d_(Q_1_-Q_3_)** | **M_d_(Q_1_-Q_3_)** |
| ***Wheezer (Case)*** | | | | |
| **IL-5** | 82 (43-166) | 65 (19-193) | 20 (8-86) | 2318 (1359-3742) |
| **IL-13** | 295 (208-561) | 49 (15-145) | 37 (14-78) | 3751 (2389-5530) |
| **IFN-g** | 168 (51-370) | 63 (18-115) | 48 (18-144) | 335 (122-953) |
| **IL-10** | 13 (8-35) | 15 (5-35) | 24 (7-52) | 310 (159-552) |
| **IL-17** | 12.5 (7-62) | 15 (7-34) | 15 (4-22) | 258 (173-388) |
| ***Non-wheezer (Control)*** | | | | |
| **IL-5** | 64 (37-140) | 70 (20-135) | 17 (8-36) | 2114 (1204-3370) |
| **IL-13** | 294 (218-475) | 45 (18-97) | 25 (14-59) | 3661 (2203-5040) |
| **IFN-g** | 113 (56-301) | 70 (24-219) | 47 (10-156) | 390 (127-921) |
| **IL-10** | 26 (11-88) | 13 (8-27) | 27 (11-66) | 288 (159-493) |
| **IL-17** | 11 (7-46) | 8 (4-13) | 14 (4-21) | 291 (185-410) |

**Table S2**: Responder rates by area of residence (urban vs. rural) and cytokine for each culture condition.

| **Stimulus/Cytokine** | **Rural Responders** | | **Urban Responders** | |
| --- | --- | --- | --- | --- |
|  | **Number of responders** | **% responders*** | **Number of responders** | **% responders*** |
| **Spontaneous Production** |  |  |  |  |
| **IL-5** | 98 | 52.13 | 144 | 62.61 |
| **IL-13** | 103 | 54.79 | 158 | 68.70 |
| **IFN-γ** | 43 | 22.99 | 84 | 36.52 |
| **IL-10** | 23 | 12.30 | 46 | 20.18 |
| **IL-17** | 13 | 6.95 | 18 | 7.86 |
| ***Ascaris lumbricoides*** |  |  |  |  |
| **IL-5** | 72 | 38.30 | 71 | 30.87 |
| **IL-13** | 97 | 51.60 | 87 | 37.83 |
| **IFN-γ** | 28 | 14.97 | 49 | 21.30 |
| **IL-10** | 29 | 15.51 | 58 | 25.33 |
| **IL-17** | 17 | 9.09 | 18 | 7.86 |
| **House dust mite** |  |  |  |  |
| **IL-5** | 36 | 19.15 | 68 | 29.57 |
| **IL-13** | 80 | 42.55 | 90 | 39.13 |
| **IFN-γ** | 32 | 17.11 | 48 | 20.87 |
| **IL-10** | 46 | 24.60 | 103 | 44.98 |
| **IL-17** | 14 | 7.61 | 23 | 10.13 |

* Overall number of observations vary by cytokine and stimulus.

**Figure S1a:** Production of IL-5 by children living in rural x urban environment; (A) spontaneous production; (B) SEB stimulation; (C) House dust mite (HDM) stimulation ; (D) *Ascaris lumbricoides* stimulation.

**Figure S1b:** Production of IL-13 by children living in rural x urban environment; (A) spontaneous production; (B) SEB stimulation; (C) House dust mite (HDM) stimulation ; (D) *Ascaris lumbricoides* stimulation.

**Figure S1c:** Production of IFN-γ by children living in rural x urban environment; (A) spontaneous production; (B) SEB stimulation; (C) House dust mite (HDM) stimulation ; (D) *Ascaris lumbricoides* stimulation.

**Figure S1d:** Production of IL-10 by children living in rural x urban environment; (A) spontaneous production; (B) SEB stimulation; (C) House dust mite (HDM) stimulation ; (D) *Ascaris lumbricoides* stimulation.

**Figure S1e:** Production of IL-17 by children living in rural x urban environment; (A) spontaneous production; (B) SEB stimulation; (C) House dust mite (HDM) stimulation ; (D) *Ascaris lumbricoides* stimulation.
